# Relation of environmental factors with activity limitations and participation restrictions in older adults with diabetes mellitus over time: an international classification of functioning framework perspective

**DOI:** 10.1186/s12877-023-03977-0

**Published:** 2023-05-30

**Authors:** Li Ai Tai, Le Yu Tsai, Yi Chen Chiu

**Affiliations:** 1 Graduate Institute of Clinical Medical Science, College of Medicine, Chang Gung University, Taoyuan, Taiwan; 2 Department of Nursing, Cardinal Tien Junior College of Healthcare and Management, New Taipei, Taiwan; 3 Department of Endocrinology and Metabolism, Yonghe Cardinal Tien Hospital, New Taipei, Taiwan; 4 School of Nursing, College of Medicine, Chang Gung University, Taoyuan, Taiwan

**Keywords:** Diabetes mellitus, International classification of functioning, Environmental factors, Activity limitations and participation restrictions

## Abstract

**Background:**

Activity limitations and participation restrictions were observed in patients with diabetes, which may impact their quality of life. Environmental factors such as seasonal effects, resources and perceived stress may play important role in activity limitations and participation restrictions. In this study, a variant of International Classification of Functioning (ICF) model was used to clarify the associations of function/structure factors, personal factors and environmental factors with activity limitations and participation restrictions.

**Methods:**

This was a longitudinal design with 1 year follow-up. The Mini-Mental State Examination (MMSE), Geriatric Depression Scale- short form, Perceived Stress Scale, and Instrumental Activities of Daily Living assessments were measured at 3-month intervals for long-term follow-up in 110 patients with diabetes aged ≥ 65 years.

**Results:**

Hierarchical linear regression models revealed that age (*p* = 0.001), perceived health status, MMSE scores (*p* < 0.01), and positive perceived stress (*p* < 0.001) were predictors of activity limitations (adjusted *R*^2^ = 53%). GDS-S (*p* < 0.05) and positive perceived stress (*p* < 0.01) were predictors of participation restrictions (adjusted *R*^2^ = 30%). Generalized estimating equation analysis indicated that seasonal effects, age, perceived health status, MMSE predicted the changes of slopes in activity limitations. Seasonal effects and GDS-S were predictors of the changes of slopes for participation restrictions (all *p* < 0.001).

**Conclusions:**

The ICF can be used to identify the risk factors for activity limitations and participation restrictions in older adults with diabetes. Practitioners should provide individualized interventions with consideration of these risk factors.

**Supplementary Information:**

The online version contains supplementary material available at 10.1186/s12877-023-03977-0.

## Background

The global diabetic population is estimated to reach 642 million by 2040 [1] which is the most common non-communicable disease globally [2], and also the most prevalent condition among older adults. Patients with diabetes were more likely to have a profound activity limitation than people without diabetes (age-standardized rates of 14%, compared with 5%). The risk of disability in patients with diabetes is 1.65 times greater in those healthy individuals [3]. The most common type of disability experienced by patients with diabetes was restriction in physical activities or work (32% of people with diabetes) [4]. Disability is a multidimensional concept with measurements that can be complex, varying across time and context [5]. Thus, the WHO proposed the International Classification of Functional, Health, and Disability (ICF) model to deal with the multidimensions of disability (ICF, 2001).

**Fig. 1:**
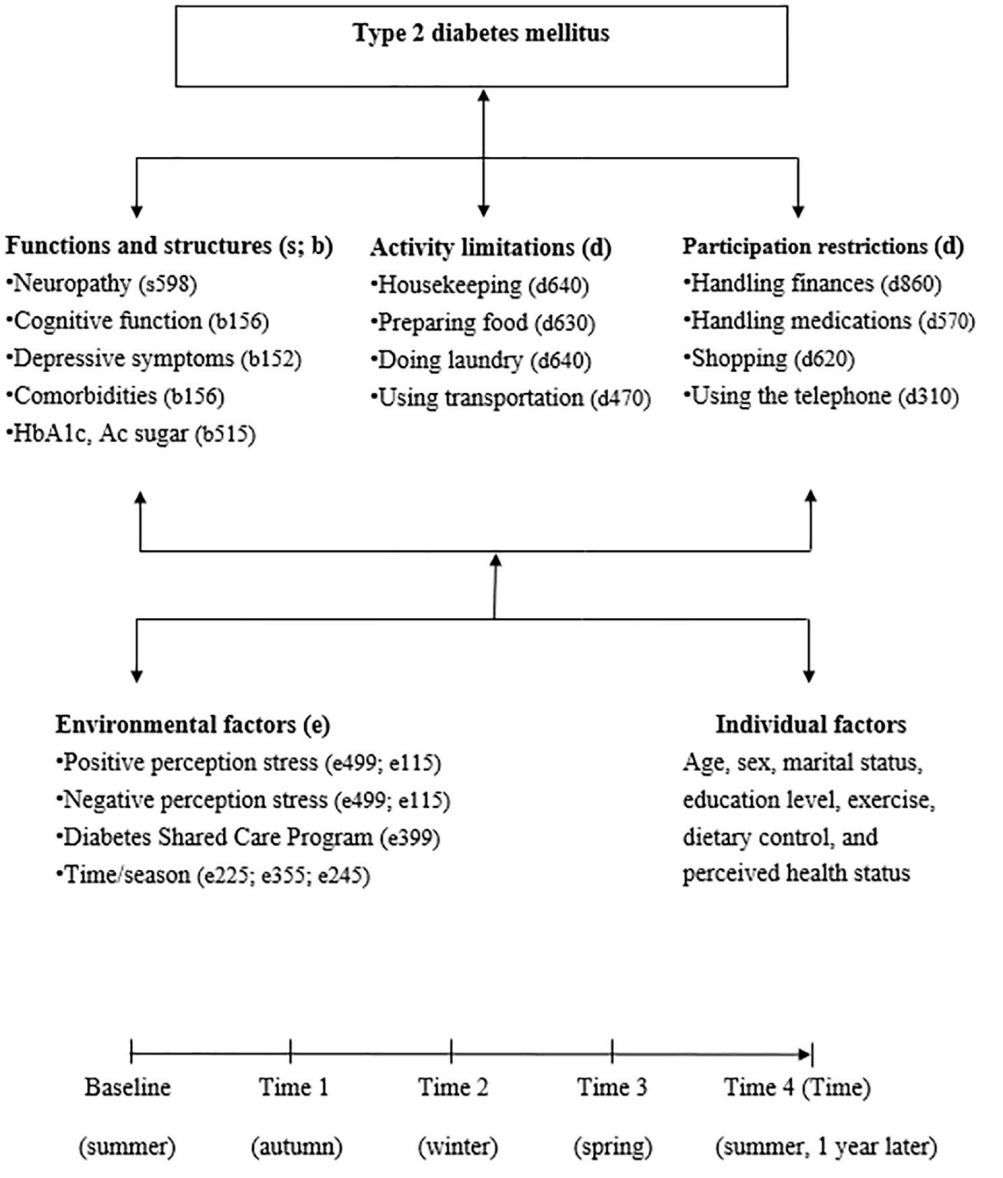
The conceptual framework was adapted from the WHO ICF and corresponding ICF codes [30, 32]

The ICF model consists of two different components [6] (Fig. 1). The first component distinguishes four concepts to operationalize disability: function/structures, activity limitations and participation restrictions.

Function and structures refer to the physiological functions and the anatomy of body systems, respectively [7]. Diabetes is a systemic chronic disease in which multiple-organ damage tends to cause high blood sugar, neuropath [7] and restrict limb mobility [8, 9]; in addition, metabolic abnormalities and comorbidities can trigger changes in physical function, thereby affect an individual’s physical activities [8–12]. Cognitive function, depression, and time are risk factors for activity limitations [13, 14]. Likewise, comorbidities and depression can interfere with interpersonal relationships and social participation behavior [8, 9, 15]. In this study, activity limitations refer to difficulties one may have when executing activities, while participation restrictions define as dealing with problems one may experience in life situations [16]. Domains of activity limitations and participation restrictions include learning, morbidity, self-care, domestic life, interactions, relationships, major life areas, and community, social and civic life. A confirmatory factor analysis of an instrumental activities of daily life instrument revealed a two-factor solution: first, domestic chores, including: washing clothes and preparing meals, and second, work and leisure, including gardening, driving and outings [17]. Therefore, we conceptualized activity limitations and participation restrictions as two different constructs based on the measurement of instrumental activities of daily life (Fig. 1).

The second component of the ICF model investigates contextual or environmental factors, at both structural and the personal level. Structural factors include support and relationships, services and policies, as well as attitudes. These factors act as facilitators of or barriers to functioning in society; personal factors include age, gender, education, and lifestyle behaviors [18], such as perceived health, regular exercise and dietary control. For a certain degree of functional impairment, the level of activity limitations and participation restrictions is variable depending on the personal and environmental factors surrounding the individual [19]. The ICF model has been applied to patients with diabetes [7] with coding systems for each component of the ICF (ICF-core sets). However, some researchers criticized that there is not a clear distinguish between activity limitations and participation restrictions. In addition, the ICF still emphasizes less on the critical role of the environment than other model, e.g., Lawton’s environmental press theory [19]. Environmental press is similar to the concept of stress, and “press” may be positive, neutral, or negative. Press can be thought of as behavioral activating to some ones, which is stimulation with motivating quality to activate a cognate individual need. Therefore, environmental press is environmental characteristic with some demand nature for the individual, whether the demand is objective or subjective. Objective environmental press or demand can be the seasonal effect, impacting behavioral pattern of patients with diabetes [20] or resources in the community such as Diabetes Shared Care Program which provides an integrated care in Taiwan and has been proven to improve the care quality of patients with diabetes [21]. Subjective environmental press deals with self-appraisal of environmental stimulations regarding its stressfulness. Perceived stress has been established to be the etiological link to diabetes, which can alter the metabolism of glucose and thus alter the glycemic status of the diabetic patients [22]. Stress has not only shown to increase the risk of diabetes but also contributes to physical inactivity and difficulties in getting rid of habits like tobacco and alcohol use [23]. Therefore, the positive or negative quality of press is dependent on whether it can elicit adaptive or nonadaptive behavior when considering individual functional level. Some researchers highlight that consideration of the ICF should also be placed on environmental factors that may be key factors to support or obstruct patient function and participation as well as independent self-care activity [24]. Conversely, maladaptation and dependence can result in a poor match between an individual function and their environment [25, 26].

In sum, disability is a complex physical and mental health matter within an environment; examining it using a theoretical framework can help clarify various constructs, especially activity limitations, participation restrictions and environmental factors. Therefore, this paper aims to employ a variant of the ICF model to explore the relationships between diabetes and disability in northern Taiwan over one year period.

## Methods

### Population and procedures

In this longitudinal observational study (project number: R106-003), we adopted convenience sampling to recruit patients with diabetes from the endocrinology outpatient clinics of hospitals in northern Taiwan with participants’ informed consent forms. We subsequently explored the illness experience of these patients. The enrollment period was June 1, 2017, to August 31, 2018. In our study framework, data of function/structure factor [27, 28], environmental factors and personal factors [8, 9, 28] were collected to predict activity limitations and participation restrictions in those with diabetes [29]. All variables were systematically linked to the most appropriate ICF components [30] and ICF Checklist [31, 32] (Fig. 1).

We performed follow-up measurements every 3 months: Baseline (summer), Time 1 (autumn), Time 2 (winter), Time 3 (spring), and Time 4 (1 year later). After the participants provided written informed consents, we conducted baseline data collection in summer, 2017. Except for participants who died, withdrew from the study, or were lost to follow-up, all those who completed follow-ups at the five time points provided self-report data; their neuropathy status, fasting blood glucose (AC sugar) and glycated hemoglobin (HbA1c) values were downloaded from electronic records.

**Fig. 2:**
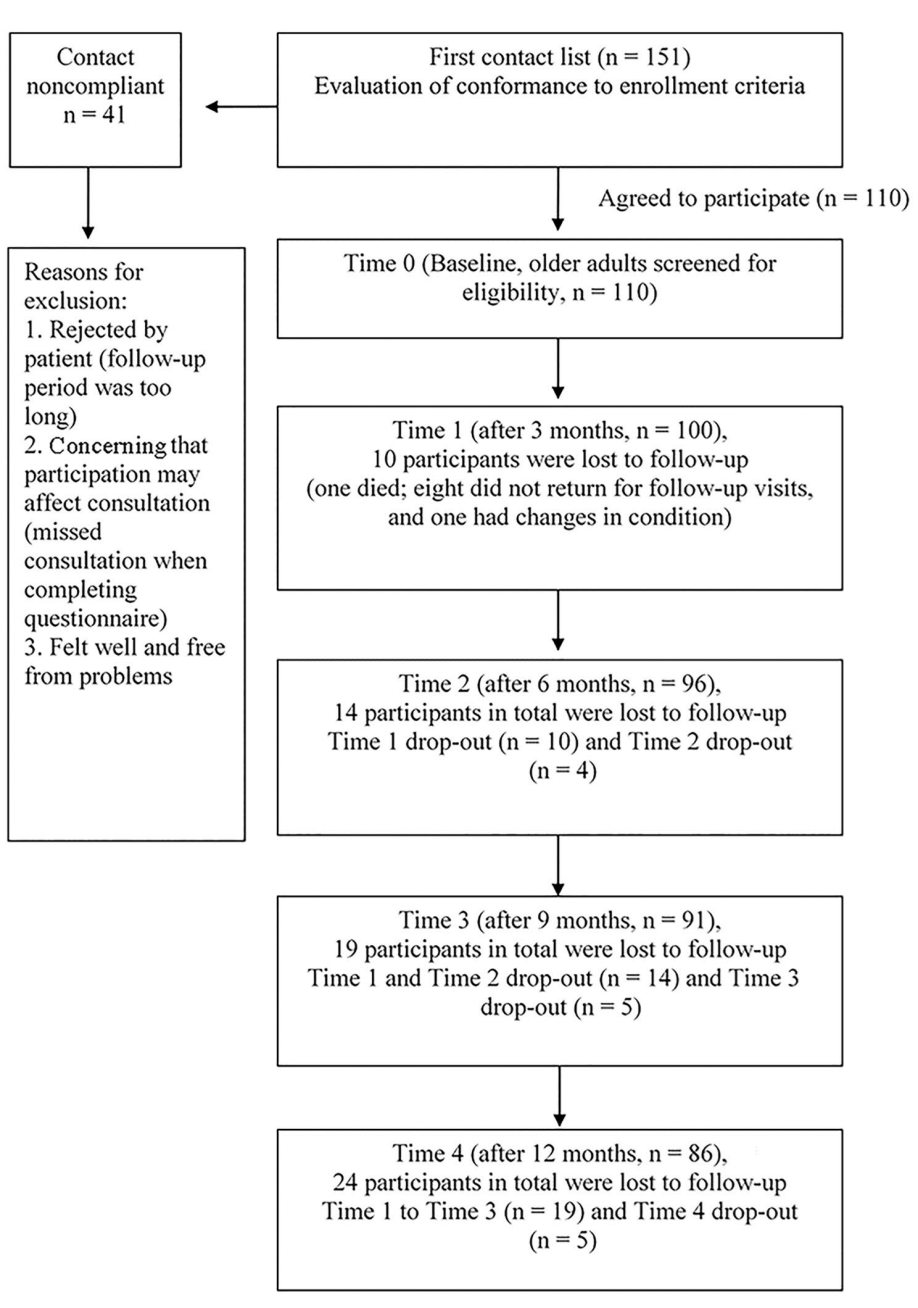
Flow diagram of the study population

G*Power 3.1 was used to determine the most appropriate sample size based on F-test linear multiple regression modeling [33]. To estimate sample size for the R² deviation from zero, we used the following settings: effect size *f*^2^ = 0.15, *α* err prob = 0.05, power (1 − *β* err prob) = 0.95, and number of measures = 5. Accordingly, the required sample size was 88. Given that our enrollment was repeated five times, that the enrollment period was 1 year, and that the crude estimated loss to follow-up rate was 20%, the estimated sample size was 105. The inclusion criteria were as follows: (1) being medically stable, by physician’s approval for participation in this study; (2) aged ≥ 65 years; (3) being able to understand and to communicate in Mandarin, Taiwanese or Hokka. Exclusion criteria: (1) terminal illness; (2) Mini-Mental State Examination (MMSE) score below 20 points [34]. A total of 110 consecutive patients with diabetes were recruited. Figure 2 presents a flow diagram of the participant selection for this study.

### Ethics

Ethical approval for this study was obtained from the human subject ethical committees of participating hospitals in northern Taiwan (the institutional review board approval number: CTH-106-3-5-011). The eligibility of the potential participants was assessed by a researcher in accordance with our inclusion criteria. Written informed consent was obtained from each eligible participant before they received a structured face-to-face interview. All interviews were conducted in private rooms in community centers to ensure confidentiality.

### Measurements

#### Dependent variables

##### Activity limitations and participation restrictions

We used a modified version of the original IADL scale developed by Lawton and Brody [35] to measure activity limitations and participation restrictions based on the 2-factor results of the exploratory factor analysis with oblique rotation (Table 1). In the modified IADL scale, the score for each item ranges between 0 and 3 points, with 0, 1, 2, and 3 indicating no difficulty, some difficulty, significant difficulty, and inability to perform, respectively; the total score thus ranges between 0 and 24 points. The reliability of the scale was 0.8–0.9 [35], test–retest reliability 0.90, and internal consistency 0.86 [36]. In this study, the Cronbach’s α coefficient for the whole IADL was 0.873. We adopted four items from the IADS scale (housekeeping, laundry, cooking, transportation), with a total combined score of 12 points, to measure activity limitations; the Cronbach’s α coefficient was 0.916. In addition, the total scores of another four items (handling finances, managing medications, shopping, using the telephone) of the IADL scale were used to evaluate participation restrictions; the Cronbach’s α coefficient for these items was 0.842. Consistent with the definitions of the corresponding ICF Core Sets categories [3, 8, 9], higher total scores on these two subscales indicate greater activity limitations or participation restrictions (Fig. 1).

**Table 1:** Principal components analysis with oblique rotation of the modified IADL scale

Items	Mean	S.D.	Factor I	Factor II
Handling finances	0.09	0.41	0.783	0.355
Housekeeping	0.89	0.94	0.138	0.888
Preparing food	0.45	0.80	0.456	0.850
Handling medications	0.05	0.25	0.814	0.225
Doing laundry	0.56	0.92	0.281	0.909
Shopping	0.10	0.45	0.799	0.287
Using transportation	0.35	0.85	0.571	0.729
Using the telephone	0.05	0.25	0.856	0.204
Extraction Sums of Squared Loadings				
Variance (%)			40.98	39.61
Cumulative (%)			40.98	80.58
Cronbach’s α			0.842	0.916
Total Cronbach’s α			0.873	

#### Independent variables

##### Personal factors

The following personal factors were considered in this study: sex, age, education level, marital status, perceived health status, and whether engaging in diabetic dietary control and performing regular exercise (more than 3 times a week). For perceived health status, we used a scale designed for older adults to indicate their health status in the past month [37, 38]. On this scale, scores range between 1 and 10 points, with a high score indicating a favorable perceived health status [38].

##### Body structural/function factors

In the participating hospital, examining HbA1c, AC sugar, and neuropathy of a patient with diabetes is a routine practice, and these indicators match with the ICF Core Sets in the function/structure domain. Therefore, we collected these data from patient’s chart within the past 3 months. In this study, effective clinical glycemic control was defined as HbA1c < 7.5% and diabetes was defined as AC sugar ≥ 200 mg/dL [39]. In addition, multiple comorbidities with systemic disease diagnosis and the total number of diseases were obtained from electronic medical records.

We used the Geriatric Depression Scale-Shortform (GDS-S) to measure depressive symptoms of patients with diabetes, which is commonly adopted to assess depression in older adults. This questionnaire has a total of 15 questions (0–15 points; *Yes* = 1 point; *No* = 0 points); a higher score indicates more severe symptoms. Studies have revealed that this scale has favorable internal consistency reliability (α = 0.94), test–retest reliability (r = 0.85; [40], and internal consistency reliability (Cronbach’s α = 0.89 [41]. The Cronbach’s α coefficient in this study was 0.80.

The Mini Mental State Examination (MMSE) is a tool to assess global cognition. This 11-question measure tests five areas of cognitive function: orientation, registration, attention and calculation, recall, and language. The maximum score is 30 [42]. The test–retest reliability supported by Pearson coefficient of the original Chinese scale is 0.87, and the Cronbach’s α is 0.91 [43]. The Cronbach’s α coefficient in this study was 0.77.

##### Environmental factors

To examine environmental factors, codes from the WHO International Classification of Functioning, Disability and Health Core Set for Diabetes Mellitus (ICF-CS for DM) [9] were selected, which matched our environmental factors. We examined three environmental factors. First, we considered “time,” which referred to the follow-up conducted in 3-month intervals to match the seasons. Second, the Chinese Perceived Stress Scale-14 item (PSS) was used; the PSS was created by Cohen et al. [44], who authorized Dr. Chu to provide a translated version. This self-rated scale measures the stress a respondent has experienced in the past month by using a five-point Likert scale (0 = *never*, 1 = *almost never*, 2 = *sometimes*, 3 = fairly *often*, and 4 = *very often*). Total score ranges between 0 and 56 points. Half of the questions are positively worded with a higher score suggesting positive perceived stress, while the other half being negatively worded with a higher score suggesting greater negative perceived stress [44]. The Cronbach’s α coefficient of the scale was 0.84–0.86, and its test–retest reliability was 0.85. The WHO definition was used as a reference in this study, and the perception stress scale was divided into positive perceived stress and negative perceived stress as facilitators and barriers in the environment, respectively (Fig. 1). The Cronbach’s α coefficients for the total PSS and the positive and negative perceived stress subscales in this study were 0.76, 0.80, and 0.71, respectively. The third and final environmental factor we considered was health care service resources: whether patients with diabetes participated in the Diabetes Shared Care Program (DSCP) (yes vs. no).

### Data analysis

Statistical analyses were exported to Microsoft Excel and then imported in IBM SPSS Statistics for Windows Version 21.0 (IBM Corp. Armonk, NY, USA) for baseline data analysis. Descriptive statistics such as frequency, percentage, mean and standard deviation described participants’ demographic characteristics. Pearson’s correlation analysis was used to evaluate the correlations between independent and dependent variables.

Independent-samples *t* tests were used to examine the differences in activity limitations and participation restrictions in each construct. We explore the related risk factors of these two dependent variables with hierarchical linear regression analysis; the regression was divided into three stages for model analysis. In the first stage, personal factors (age, education level, regular exercise, dietary control, and perceived health status) were tested. In the second stage, function/structure factors (neuropathy, HbA1c, Ac sugar, MMSE scores, depression) were tested. In the third stage, environmental factors (positive and negative perception stress, DSCP participation), and missing sample were also controlled. Missing sample refers to participants who did not attend interviews after the first one for whatever reason. R^2^, modified R^2^, and regression coefficients (*β*) were used to assess each prediction model [45], and the predictors affecting activity limitations and participation restrictions in patients with diabetes were examined. Finally, the generalized estimating equation (GEE) AR 1 statistical method was used to analyze whether changes in different dimensional influencing factors would have different effects on activity limitation and participation restriction. The significance level for all tests was set at an alpha level of 0.05.

## Results

### Exploratory factor analysis

A Principal Components Analysis with oblique rotation was used to explore the factor structure of the IADL items using the baseline data of 110 participants [46]. Table 1 presents the item means and standard deviations and factor loadings for the two-factor solution. The first factor (participation restrictions) consisted of 4 items with explained variance of 41.0%, and Cronbach’s α 0.84. The second factor (activity limitations) consisted of 4 items with explained variance of 39.6%, and Cronbach’s α (0.92). The cumulative explained variance was 80.6%, while the total Cronbach’sαwas 0.87.

### Characteristics of the studied population

At baseline, the mean age was 73.43 years (SD = 6.91), and most of the participants were women (61.8%). In total, 47.3% exercised regularly, and 51.8% performed diabetic dietary control. The mean score for perceived health status was 6.24 points (SD = 1.63). The mean number of chronic comorbidities was 2.75 (SD = 1.14), and 36.4% of the participants had neuropathy. The DSCP participation rate was 67.3% (Table 2).

**Table 2:** Clinical characteristics of patients with diabetes (baseline, n = 110)

Characteristics	Number (%)	Mean (± SD)
Personal characteristic factors		
Age		73.43 (6.91)
Female sex	68 (61.8)	
Years of education		9.06 (4.18)
Regular exercise (yes)	52 (47.3)	
Diabetic dietary control (yes)	57 (51.8)	
Perceived health status (1–10 points)		6.24 (1.63)
Structural/functional factors		
Neuropathy (yes)	40 (36.4)	
Multiple comorbidities		2.75 (1.14)
2 or fewer diagnoses	62 (56.4)	
>2 diagnoses	48 (43.6)	
MMSE (1–30 points)		23.34 (3.15)
GDS-S (0–15 points)		3.94 (3.27)
HbA1c		7.82 (1.49)
AC Sugar		149.50 (58.70)
Environmental factors		
PPS (0–28 points)		18.34 (4.68)
NPS (0–28 points)		10.48 (4.12)
DSCP	74(67.3)	
Activity limitations (0–12 points)		2.08 (3.32)
Participation restrictions (0–12 points)		0.45 (1.52)

**Abbreviations**: a: MMSE, Mini-Mental State Examination; GDS-S, Geriatric Depression Scale- short form; HbA1c, glycated hemoglobin; PPS, positive perceived stress; NPS, negative perceived stress; DSCP, Diabetes Shared Care Program

### Factors influencing activity limitations and participation restrictions

The factors influencing activity limitations were not conducting irregular exercise (mean = 2.88; SD = 3.93), having neuropathy (mean = 3.30; SD = 3.91), and missing samples from the study (mean = 4.32; SD = 4.13), all yielding significant differences in activity limitations. As to participation restrictions, not participating in dietary control (mean = 0.75, SD = 2.01), having neuropathy (mean = 0.95, SD = 2.33), missing samples from the study (mean = 1.28; SD = 2.46) all yielded significant differences in this dependent variable. (Table 3).

**Table 3:** Factors associated with activity limitations and participation restrictions in patients with diabetes

Variable			Activity limitations				Participation restrictions		
	Group	n	Mean (SD)	*t* value	*p* value		Mean (SD)	*t* value	*p* value
Sex	Female	68	2.03 (3.11)	−0.21	0.834		0.46 (1.56)	0.09	0.927
	Male	42	2.17 (3.20)				0.43 (1.47)		
Regular exercise	No	58	2.88(3.93)	2.82	0.006		0.66 (1.75)	1.57	0.119
	Yes	52	1.19 (2.20)				0.21 (1.18)		
Dietary control	No	53	2.72 (3.57)	1.95	0.054		0.75 (2.01)	2.04	0.046
	Yes	57	1.49 (2.98)				0.16 (0.75)		
Neuropathy	No	70	1.39 (2.73)	−2.74	0.008		0.16 (0.58)	−2.12	0.041
	Yes	40	3.30 (3.91)				0.95 (2.33)		
Multiple comorbidities	No	62	1.65 (2.98)	−1.74	0.085		0.23 (0.90)	−1.60	0.116
	Yes	48	2.65 (3.67)				0.73 (2.04)		
DSCP	No	36	3.17 (3.73)	-1.54	0.118		0.86 (2.28)	-1.60	0.116
	Yes	74	1.55 (2.99)				0.24 (0.90)		
Missing samples	No	85	1.42 (2.74)	−3.30	0.002		0.20 (1.00)	−2.45	0.041
	Yes	25	4.32 (4.132)				1.28 (2.46)		

a: Missing samples was defined as any absent record in the one-year follow-up period from baseline to 12 months of follow-up

### Correlation analysis of activity limitations and participation restrictions

The activity limitations and participation restrictions of our sample are increased with age (*r* = 0.51, *r* = 0.39, respectively). Activity limitations are increased with poor perceived health status (*r* = − 0.26), and lower MMSE (*r* = − 0.44). Higher activity limitations and participation restrictions are associated more severe GDS-S (*r* = 0.34, *r* = 0.39, respectively), and lower PPS (*r* = − 0.54, *r* = − 0.46, respectively), and higher NPS (*r* = 0.37, *r* = 0.19, respectively) (Table 4).

**Table 4:** Results of the ICF framework: diabetes with activity limitations and participation restrictions

Variable	Age	Education	Health status	MMSE	GDS-S	Multiple comorbidities	HbA1c	AC Sugar	PPS	NPS	Activity limitations	Participation restrictions
Age	1											
Education level	− 0.12	1										
Health status	0.05	0.19^*^	1									
MMSE	− 0.34^**^	0.30^**^	0.04	1								
GDS-S	0.22^*^	− 0.01	− 0.19^*^	− 0.20^*^	1							
Multiple comorbidities	0.05	− 0.09	− 0.08	− 0.14	− 0.01	1						
HbA1c	− 0.03	− 0.14	− 0.03	− 0.17	− 0.05	0.03	1					
AC Sugar	− 0.03	− 0.18	0.02	− 0.16	− 0.02	0.11	0.59^**^	1				
PPS	− 0.38^**^	0.05	0.03	0.25^**^	− 0.36^**^	− 0.03	0.01	− 0.05	1			
NPS	0.24^*^	− 0.03	− 0.32^**^	− 0.23^*^	0.48^**^	− 0.07	− 0.02	− 0.04	− 0.23^*^	1		
Activity limitations	0.51^**^	− 0.17	− 0.26^**^	− 0.44^**^	0.34^**^	0.02	0.05	− 0.00	− 0.54^**^	0.37^**^	1	
Participation restrictions	0.39^**^	− 0.06	− 0.18	− 0.17	0.39^**^	− 0.07	0.05	0.01	− 0.46^**^	0.19^*^	0.65^**^	1

**Abbreviations**: a: MMSE: Mini-Mental State Examination; GDS-S: Geriatric Depression Scale- short form; PPS: Positive perceived stress; NPS: negative perceived stress

*p* < 0.05, ^**^p < 0.01

### Environmental factors mediating predictors of activity limitations and participation restrictions in patients with diabetes

The significant associated factors in Tables 3 and 4 are selected and entered hierarchical liner regression models to identify significant predictors and mediators for activity limitations and participation restrictions. As to activity limitations, Level I shows: age *β* (95% CI) = 0.54 (0.19, 0.33), regular exercise *β* (95% CI) = − 0.24(− 2.59, − 0.55), and perceived health status *β* (95% CI) = − 0.23(− 0.79, − 0.16). In Level II, after function/structure factors were added, the significant predictors were age *β* (95% CI) = 0.39(0.11, 0.26), regular exercise *β* (95% CI) = − 0.19 (− 2.22, − 0.27), perceived health status *β* (95% CI) = − 0.21 (− 0.73, − 0.14), and MMSE scores *β* (95% CI) = − 0.25 (− 0.43, − 0.11). In Level III, after environmental factors were added, the significant predictors were age *β* (95% CI) = 0.26 (0.05, 0.20), perceived health status *β* (95% CI) = − 0.20 (− 0.70, − 0.11), MMSE scores *β* (95% CI) *= −* 0.22 (− 0.38, − 0.08), and PPS *β* (95% CI) = − 0.29 (− 0.31, − 0.10) (Table 5). The final Level explained 53% of the adjusted variance of activity limitations.

**Table 5:** Hierarchical linear regressions prediction of activity limitations, participation restrictions in patients with diabetes

Activity limitations	Level I β (95% CI)	Level II β (95% CI)	Level III β (95% CI)
**Personal factor**			
Age	0.54^***^(0.19, 0.33)	0.39^***^(0.11, 0.26)	0.26^**^(0.05, 0.20)
Regular exercise (yes)	-0.24^**^(-2.59, -0.55)	-0.19^*^(-2.22, -0.27)	-0.12(-1.72, 0.17)
Perceived health status	-0.23^**^(-0.79, -0.16)	-0.21^**^(-0.73, -0.14)	-0.20^**^(-0.70, -0.11)
**Function/structure factors**			
Neuropathy (yes)		0.12(-0.18, 1.84)	0.13(-0.04, 1.86)
MMSE		-0.25^**^(-0.43, -0.11)	-0.22^**^(-0.38, -0.08)
GDS-S		0.11(-0.04, 0.26)	0.01(-0.16, 0.17)
**Environmental factors**			
PPS			-0.29^***^(-0.31, -0.10)
NPS			0.08(-0.07, 0.19)
Missing samples (yes)			0.08(-0.56, 1.79)
*R* ^2^	0.40	0.49	0.56
Adjusted *R*^2^	0.38	0.46	0.53
Increase in *R*^2^	0.40	0.09	0.07
F Change	23.17^***^	6.09^**^	5.88^**^
**Participation restrictions**	**Level I β (95% CI)**	**Level II β (95% CI)**	**Level III β (95% CI)**
**Personal factors**			
Age	0.37^***^(0.04, 0.12)	0.28^**^(0.02, 0.10)	0.17(-0.01, 0.08)
Dietary control (yes)	-0.16(-1.02, 0.04)	-0.09(-0.81, 0.25)	-0.10(-0.80, 0.21)
**Function/structure factors**			
Neuropathy (yes)		0.11(-0.23, 0.90)	0.11(-0.20, 0.88)
GDS-S		0.29^**^(0.06, 0.22)	0.22^*^(0.01, 0.19)
**Environmental factors**			
PPS			-0.28^**^(-0.15, -0.03)
NPS			-0.04(-0.08, 0.05)
Missing samples (yes)			0.09(-0.32, 0.95)
*R* ^2^	0.17	0.27	0.35
Adjusted *R*^2^	0.16	0.24	0.30
Increase in *R*^2^	0.17	0.93	0.79
F Change	11.28^***^	6.69^**^	4.10^**^

**Abbreviations**: a: MMSE, Mini-Mental State Examination; GDS-S, Geriatric Depression Scale- short form; PPS, Positive perceived stress; NPS, Negative perceived stress

^*^p < 0.05, ^**^p < 0 0.01, ^***^p < 0.001

As to participation restrictions, the significant predictors in Level I were age *β* (95% CI) = 0.37 (0.04, 0.12). In Level II, after function/structure factors were added, the significant predictors were age *β* (95% CI) = 0.28 (0.02, 0.10) and GDS-S score *β* (95% CI) = 0.29 (0.06, 0.22). In Level III, after environmental factors were added, the significant predictors were GDS-S score *β* (95% CI) = 0.22 (0.01, 0.19), and PPS *β* (95% CI) = − 0.28 (− 0.15, − 0.03; Table 5). The final Level explained 30% of the adjusted variance in participation restrictions.

### The predictors of changes in the slopes for activity limitations and participation restrictions in patients with diabetes

Over the four seasonal time points, the mean score for activity limitations ranged from 2.04 (SD = 2.94) to 2.47 (SD = 3.15), with activity limitations being the most severe in spring. Activity limitations in the summer of the following year was still higher than that at baseline (summer), whereas participation restrictions were attenuated [see Additional file 1]. As to activity limitations, the environmental factors, such as effects of autumn *β* (95% CI) = − 0.16 (− 0.25, − 0.07), winter *β* (95% CI) = 0.26 (0.19, 0.33), spring *β* (95% CI) = 0.38 (0.32, 0.45), and summer the following year *β* (95% CI) = 0.29 (0.24, 0.35) as well as PPS *β* (95% CI) = − 0.11 (− 0.16, − 0.06) were statistically significant predictors of the changes of slopes in activity limitations. Age in personal factors *β* (95% CI) = 1.19 (1.03, 1.34), perceived health status *β* (95% CI) = − 0.46(− 0.52, − 0.39), and function/structure factor MMSE *β* (95% CI) = − 0.25 (− 0.28, − 0.23) were still predictors of slops of changes in activity limitations. The predictors of exposures were the same in both the final hierarchical linear regression model and the GEE model for activity limitations. As to participation restrictions, the GEE analysis revealed that the environmental factors such as effects of autumn *β* (95% CI) = − 0.21 (− 0.26, − 0.16), winter *β* (95% CI) = − 0.22 (− 0.26, − 0.17), spring *β* (95% CI) = − 0.27 (− 0.31, − 0.22), and summer the following year *β* (95% CI) = − 0.30 (− 0.34, − 0.27) were statistically significant predictors of the changes of slopes in participation restrictions. Function/structure factor GDS-S β (95% CI) = 0.14 (0.11 − 1.16) was also the statistically significant predictor of the changes of slopes in participation restrictions (Table 6).

**Table 6:** GEE analysis of predictors of the changes for activity limitations and participation restrictions in patients with diabetes

Variable	Activity limitations					Participation restrictions			
	*B*	*SE*	95% C I	*p* value		*B*	*SE*	95% C I	*p* value
(Intercept)	12.34	0.83	10.70, 13.97	< 0.001		0.26	0.24	-0.20, 0.73	0.264
**Personal factors**									
Aged ≥ 75 years	1.19	0.08	1.03, 1.34	< 0.001					
Perceived health status (1–10 points)	-0.46	0.03	-0.52, -0.39	< 0.001					
**Function/structure factors**									
MMSE (1–30 points)	-0.25	0.01	-0.28, -0.23	< 0.001					
GDS-S (0–15 points)						0.14	0.01	0.11, 0.16	< 0.001
**Environmental factors**									
PPS	-0.11	0.03	-0.16, -0.06	< 0.001		-0.02	0.01	-0.05, 0.01	0.139
Time (season)									
Time 4 (summer, 1 year later)	0.29	0.03	0.24, 0.35	< 0.001		-0.30	0.02	-0.34, -0.27	< 0.001
Time 3 (spring)	0.38	0.03	0.32, 0.45	< 0.001		-0.27	0.02	-0.31, -0.22	< 0.001
Time 2 (winter)	0.26	0.04	0.19, 0.33	< 0.001		-0.22	0.02	-0.26, -0.17	< 0.001
Time 1 (autumn)	-0.16	0.05	-0.25, -0.07	< 0.001		-0.21	0.03	-0.26, -0.16	< 0.001
Time 0 (summer) ref.									

a: Reference: baseline (summer); Age (< 75 years)

b: baseline (summer); Time 1 (autumn); Time 2 (winter); Time 3 (spring); Time 4 (summer 1 year later)

## Discussion

The present results indicate that the ICF model can be applied as a common language to understand disability all over the world. It serves as a framework to conceptualize disability and how human functioning related to function/structure factors, environmental factors, and personal factors influence activity limitations and participation restrictions. It can help to clarify the risk factors of activity limitations and participation restrictions in those with diabetes as well as to predict the changes of slopes in these two dependent variables over 1 year period. Since ICF Core Sets have been published for use in patients with diabetes, several researchers pointed out that the focus of its application were still on medical aspects of disability [47]. Therefore, we emphasize activity limitations and participation restrictions and observed that activity limitations were getting worse, while participation restrictions were attenuated within 1 year period in those with diabetes. Empirical evidence shows that in patients with diabetes, cognitive function deteriorates by approx. 20% within 20 years [48], which can be a possible reason why activity limitations increase over time in our study since these two factors are significantly correlated with each other in our study and other research [49]. As to participation restrictions being attenuated within a short follow-up within 1 year, it is possible that patients with diabetes are adjusting their disease by applying assistance devises to help them participating in their social life. However, detailed related information with a longer follow-up are needed to figure out the possible explanation of the changes of activity limitations and participation restrictions.

The function/structure factors such as HbA1c and Ac sugar have no significant effects on activity limitations and participation restrictions. It could be that such conditions are still in the early stages of diabetes without apparent symptomatic impacting on these two dependent variables [47]. However, we observed that the risk factors on activity limitations include personal factors, such as age and perceived health status, as well as function/structure factors: MMSE scores, and environmental factor such as seasonal effects and positive perceived stress. The same risk factors also predict the changes of slopes of activity limitations in patients with diabetes over 1 year. The effects of risk factors on participation restrictions include function/structure factors: GDS-S and environmental factors such as seasonal effects and positive perceived stress. However, only positive perceived stress and seasonal effects predict the changes of slopes in participation restrictions over 1 year. It has been reported that older people were more likely to experience activity limitations [28], This has been reported in research conducted in Western countries [49], however, we also observe the age effect on activity limitations and participations in Taiwan. There are also strong associations between aging, activity limitations and function/structure factors such as with decreased cognitive function [49]. Cognitive function is a major component when it comes to self-management or preventing one-self from serious harm specially in patients with diabetes. These patients are required to conduct regular follow-up, self-care, adherence to diet, exercise, and medications and that all depend mainly on an intact memory [50]. Therefore, future research should examine the specific memory function and two dependent variables in patients with diabetes. In the present study, the perceived health status of the participants was related to activity limitations. This result is supported by previous research [51] and call for intervention development targeting on cognitive function of patients with diabetes regarding self-care activities.

On the other hand, depressive symptoms are associated with participation restrictions and predict their changes of slopes over 1 year. Elevated depressive symptoms do not necessarily indicate the presence of a psychiatric disorder and may be more reflective of emotional distress related to various life stressors [52], such as emotional distress related specifically to the burden of living with diabetes and its management, emphasizing the situational context of diabetes to explain the occurrence of distress. Therefore, positive outlook or positive perceived stress in the environmental factors becomes critical when living with diabetes, which involving perceived success in dealing with irritating life hassles, effectively coping with important changes occurring in participant’s life [53]. When designing future research directions targeting on improve social participation, effort must be focused on improving depressive symptoms and enhancing positive stress as a facilitator, to deal with diabetes distress.

Finally, our findings indicate another environmental factor, seasonal effects, are also risk factors for activity limitations and participation restrictions and predict their changes of slopes within 1 year. Studies have indicated that seasonal changes are associated with specific circulatory disorders and limb weakness [54] and have varying physiological, psychological, and social function effects on patients over time [55]. Studies have revealed that environmental factors affect activity limitations and physical and mental impairment [56] and that participation restrictions in 65-year-olds are related to temperature, especially seasonal temperature changes from September to January [54]. In the future, patients with diabetes should be regularly evaluated for changes in activity limitations and participation restrictions to prevent them from further physical activity dysfunction. The ICF model can be used to identify risk factors of activity limitations and participation restrictions, including age, perceived health status, cognitive function, and depression as well as positive perceived stress and seasonal changes over time. Therefore, comprehensive assessment and self-management interventions can tailor to these factors.

Our study has some limitations. First, we did not objectively evaluate neuropathy, cognitive function, limb mobility and tension, metabolic and physiological markers, and changes in physical activity. Second, we did not enroll hospitalized diabetic patients, who may be severely disabled. In the future, studies should consider the time of diagnosis, type of comorbidity, intrinsic and extrinsic resources in the long-term follow-up. Based on these information, tailored intervention studies can be conducted to evaluate the intervention effectiveness on quality of life in those with diabetes.

## Conclusion

To the best of our knowledge, this is one of the first studies to investigate the determinants of activity limitation and participation restrictions using a variant of the ICF model in those with type 2 diabetes mellitus over time. Another strength of our study is to identify the environmental factors, including positive perceived stress and seasonal effects on activity limitations and participation restrictions, which lay out a new direction for further intervention developments on comprehensive assessment and tailored strategies for helping patients with diabetes.

## Electronic supplementary material

Below is the link to the electronic supplementary material.

Supplementary Material 1

## Data Availability

The datasets used and/or analysed during the current study are available from the corresponding author upon reasonable request.
