# Supplementary material for: Relation of environmental factors with activity limitations and participation restrictions in older adults with diabetes mellitus over time: an international classification of functioning framework perspective
Source: BMC Geriatr. 2023 May 30;23:335. doi: 10.1186/s12877-023-03977-0 (PMC10227971; doi:10.1186/s12877-023-03977-0)
Supplement: Supplementary file 1 — Supplementary Material 1 [file 12877_2023_3977_MOESM1_ESM.docx]

**Appendix 1** The change of activity limitations and participation restrictions in diabetes over 1 year

| **Outcome/Time** | **Baseline**  **(summer)**  **(n = 110)** | **Time 1**  **(autumn)**  **(n = 100)** | **Time 2**  **(winter)**  **(n = 96)** | **Time 3**  **(spring)**  **(n = 91)** | **Time 4**  **(summer, 1 year later; n = 86)** |
| --- | --- | --- | --- | --- | --- |
| Activity limitations (0–12 points; mean ± SD) | 2.08  (3.32) | 2.04  (2.94) | 2.39  (3.09) | 2.47  (3.15) | 2.33  (3.21) |
| Participation restrictions (0–12 points; mean ± SD) | 0.45  (1.52) | 0.29  (1.39) | 0.27  (1.08) | 0.24  (0.93) | 0.19  (0.93) |

This Appendix 1: Summary of the the mean score for activity limitations, and participation restrictions. The following table summarises the findings of the over the four seasonal time points (baseline to 1 year later).
